# Experimental and Numerical Investigations of High-Speed Projectile Impacts on 7075-T651 Aluminum Plates

**DOI:** 10.3390/ma12172736

**Published:** 2019-08-26

**Authors:** Jae-Wook Jung, Sang Eon Lee, Jung-Wuk Hong

**Affiliations:** Department of Civil and Environmental Engineering, Korea Advanced Institute of Science and Technology, Daejeon 34141, Korea

**Keywords:** failure strain, high-speed impact, gas-gun system, finite element method, 7075 aluminum alloy

## Abstract

Simulation of the material failure under high strain rate conditions is one of the most difficult problems in the finite element analyses, and many researchers have tried to understand and reproduce dynamic material fracture. In this study, we investigate a failure criterion that minimizes the mesh dependency at high strain rates and incorporates the criterion into the Johnson-Cook constitutive relationship by developing a user-defined material model. Impact tests were performed using a gas-gun system in order to investigate the response of the 7075-T651 aluminum plate in high-speed collision. On the other hand, numerical simulations are carried out by considering various element sizes and the relationship between element size and failure strain is inversely obtained using numerical results. By accommodating the relationship into the damage model and implementing in the user-defined material model, mesh dependency is significantly reduced, and sufficient accuracy is achieved with alleviated computational cost than the existing damage model. This study suggests an element size-dependent damage criterion that is applicable for impact simulation and it is expected that the criterion is useful to obtain accurate impact responses with a small computational cost.

## 1. Introduction

An impact is defined as a mechanical process that involves the collision of two or more bodies. The relevant engineering has a wide range of applications, such as the safety assessment of buildings and nuclear reactor vessels, the assessment of the crashworthiness of vehicles, the protection of cargo and barriers, and the design of military vehicles and armor systems. As opposed to static or conventional dynamic loading, forces created by collisions are exerted and removed in an extremely short time duration. Penetration is described as the entrance of an object into a target body without passing through the body, resulting in the embedment of the striker and the formation of a crater, whereas perforation is defined as the complete escape of the target after impact.

Numerous experimental and numerical studies on impact phenomenon have been conducted by many researchers. Backman and Goldsmith studied a comprehensive survey of the mechanics of penetration of projectiles into targets [1]. An empirical formula for determining projectile penetration into steel barriers is proposed and a method for determining the ballistic limit for penetrating a target from penetration depth is presented [2]. Johnson et al. conducted research on the quasi-static piercing of metal plates [3,4]. Corbett et al. researched the penetration and perforation of plates and cylinders by free-flying projectiles traveling at sub-ordnance velocities [5]. A numerical analysis of the ballistic perforation of an impactor through steel plates was performed by Lee et al. using the peridynamic method [6]. Garcia et al. investigated the impact behavior of polymer composites [7] and Garcia and Trendafilova proposed an approach to predict the damage caused by impact loads in composite structures [8]. In addition to ballistic impacts, research on aircraft impact that can cause significant damage has also been actively conducted since the 9/11 terrorist attacks. Lee et al. conducted an evaluation of the collision of commercial aircraft [9], as well as the military aircraft impact [10].

The dynamic behavior of materials is different from the static behavior because the stiffness and inertia of the material affects the mechanical behavior. In particular, the yield stress and hardening phenomenon depend on the strain rate. To implement the effect of the strain rate in numerical material models, various studies have been carried out. One of the techniques to apply the strain rate effect to the material model is controlling the properties of the plastic region of the material. To apply the strain rate effect to the material model, the technique of scaling the plastic range by the strain rate is employed [11,12]. The Johnson-Cook material model introduces the dimensionless parameter of the strain rate to determine the plastic behavior of materials and the behavior of the plastic region is determined by the strain rate [11]. For performing the impact or blast failure where large deformation occurs during a short period, it is essential to describe the material failure. The material model is used together with the erosion algorithm for describing material failure [13], and the failure strain is used as a representative failure criterion. It is essential to select the appropriate value of failure strain in order to perform numerical analyses accurately. However, various failure strain values have been applied in other studies [14,15], which means that the failure strain values vary depending on the researcher or numerical model. Therefore, the failure strain is not fixed to a specific value but has various values depending on the analysis conditions, such as the strain rate and the element size. The failure strain is known to be dependent on the element size of the finite element model [15,16] and small changes in the failure strain cause a substantial effect on the numerical result [17]. The effect of element size on failure criterion has been investigated in various fields. Alsos et al. investigated the influence of the element size on the failure strain for finite element analysis about resistance to the penetration of stiffened plates [18]. Gang and Kwak studied the failure criterion that can minimize mesh dependency of blast simulation on reinforced concrete [19]. Villavicencio et al. confirmed the dependence of the failure strain on the element size for impact simulation on circular aluminum plates [20]. Grag and Abolmaali performed numerical analyses by varying the failure criterion according to the element size in the analysis of the reinforced concrete box culverts [21] and Ehlers applied element length-dependent failure strain in fracture of the thin circular plate [22]. Raimondo et al. proposed a mesh size independent failure model in the impact analysis of composite laminates [23].

The 7000 series of aluminum alloys, which are investigated in this study have various advantages, such as being lightweight, having high strength, and an excellent machinability, and are used in various fields, such as the commercial, military, and aviation sectors [24,25]. 7075 aluminum alloy is the highest strength alloy of aluminum, with high fracture toughness and low fatigue crack growth. The alloy is durable with a strength comparable to steel and has an excellent fatigue strength and machinability. However, 7075 aluminum alloy has a lower resistance to corrosion than many other aluminum alloys. The first 7075 was developed in secret by a Japanese company, Sumitomo Metal, in 1943 [26]. 7075 was eventually used for airframe production in the Imperial Japanese Navy.

In this study, we propose a damage criterion that minimizes mesh dependency and enables efficient impact simulations. We implement the Johnson-Cook constitutive relationship in the user-defined material model in LS-DYNA and applied the criterion in the subroutine of the material model. Numerical simulations are conducted to observe the effects of element size and failure strain on the perforation response depending on the strain rate. Experimental studies on high-velocity impact against the 7075-T651 aluminum target were carried out. In high-speed impact tests, projectiles were propelled at different initial velocities onto the aluminum plates of two different thicknesses. By comparing the residual velocity after perforation with the test results, the element size and failure strain yielding the same residual velocities with experiment results are inversely calculated. Consequently, the relationship between the element size and failure strain is applied to the damage model, and we verify the efficiency and accuracy of the damage model.

## 2. Material Model

### 2.1. Johnson-Cook Material Model

The aluminum plate is constructed using the simplified Johnson-Cook model. The Johnson-Cook material model represents the constitutive relationship for metals and is widely used to describe the dynamic behavior of the materials, such as impact and penetration. The advantage of this material model is that it is relatively easy to determine the material constants [27]. In addition, the Johnson-Cook model has been applied to various commercial finite element analysis software because of its low computational cost due to the simple form [28]. The flow stress $\sigma_{y}$ of the model is expressed as:

(1) $$\sigma_{y}\;=\;\left(a\;+\;b{\bar{\varepsilon }}^{p}{}^{n}\right)\left(1\;+\;c\ln{\dot{\varepsilon }}^{*}\right)\left(1\;-\;T^{*}{}^{m}\right)$$

where *a*, *b*, *c*, *n*, and *m* are the user-defined parameters, ${\bar{\varepsilon }}^{p}$ is the effective plastic strain, $\dot{\varepsilon^{*}}$ is the effective plastic strain rate, and $T^{*}$ is defined as $T^{*}\;=\;\left(T\;-\;T_{\mathrm{room}}\right)/\left(T_{\mathrm{melt}}\;-\;T_{\mathrm{room}}\right)$ [29]. The parameter *m* is ignored in the simplified Johnson-Cook model. If excessive deformation occurs during the finite element analysis, the analysis becomes unstable, or the computational cost significantly increases. To solve this problem, a technique of removing the elements with excessive deformation has been used, and the failure strain is used as one of the criteria for the removal. The failure strain $\varepsilon^{f}$ in the Johnson-Cook model is expressed by:

(2) $$\varepsilon^{f}\;=\;\left(D_{1}\;+\;D_{2}\exp D_{3}\sigma^{*}\right)\left(1\;+\;D_{4}\ln{\dot{\varepsilon }}^{*}\right)\left(1\;+\;D_{5}T^{*}\right)$$

where $D_{i}$ are constants and $\sigma^{*}$ is the triaxiality of stress.

### 2.2. Element Dependent Failure Strain

During the collision, the kinetic energy of the projectile is transferred to the strain energy of the target, and the projectile proceeds with the residual kinetic energy in the perforation failure mode. Therefore, the factors influencing the strain energy transfer to the target affect the residual velocity of the projectile. The failure strain affects the strain energy transferred. The reason is that the strain energy accumulates until the strain of the element reaches the failure strain. Also, the size of the element affects the volume of the removed elements and strain energy of the target. In order to achieve a high accuracy for the simulation, sufficiently small elements and a substantial computational cost are essential. However, there is a limitation on the applicable element size according to the simulation scale and, therefore, the appropriate value of the failure strain should be determined by the element size. In this study, we propose a failure strain criterion incorporating the impact velocity and element size (element dependent failure strain: EDFS) to accurately evaluate the impact response regardless of changes in element size as:

(3) $$\varepsilon^{f}\;=\;v^{*}\left(e^{v^{*}}\;-\;1\right)e^{-h^{*}}$$

where e is Euler’s number, $v^{*}$ is a dimensionless parameter of the initial velocity, and $h^{*}$ is a dimensionless parameter of the element size. The initial velocity $v_{i}$ and the element size h are normalized by the reference velocity $v_{\mathrm{ref}}$ and the reference size $h_{\mathrm{ref}}$, respectively, as $v^{*}\;=\;v_{\mathrm{ref}}/v_{i}$, and $h^{*}\;=\;h/h_{\mathrm{ref}}$.

### 2.3. User Defined Material Model (UMAT) for LS-DYNA

The material model for impact simulation has been implemented into LS-DYNA, a commercially explicit dynamic finite element code. LS-DYNA has a UMAT option where a user can implement a new material model as a subroutine. The Johnson-Cook constitutive relationship is implemented in UMAT for LS-DYNA in order to incorporate the damage criterion with the Johnson-Cook constitutive relationship. At each time step, the equation of motions for the dynamic system are calculated at the integration points of each element. The strain increment is determined by the calculated displacement of the node and is used as the variable for the UMAT subroutine. The stress increment at each time step is calculated using the stress update algorithm according to the strain increment. In this process, the internal variables of the constitutive model are also updated.

## 3. Impact Test Using Gas-gun System

### 3.1. Experimental Setup

**Figure 1:**
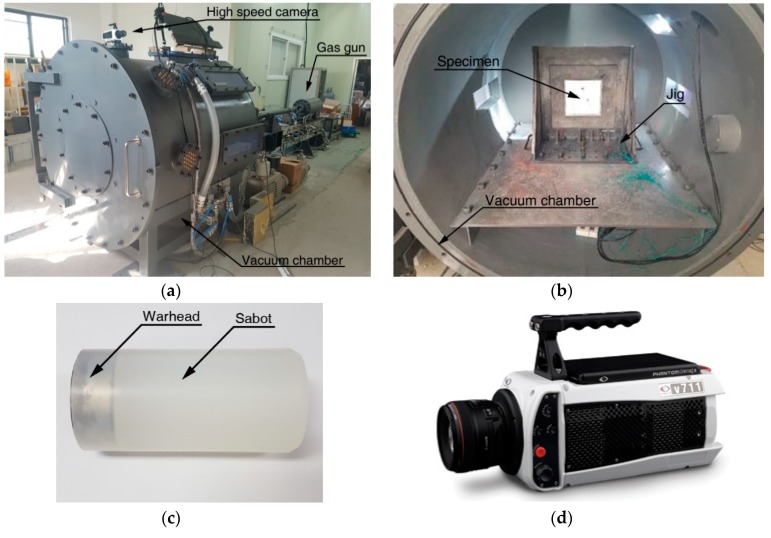
(**a**) Gas-gun system, (**b**) vacuum chamber, (**c**) projectile set, and (**d**) high-speed camera (Phantom V711).

A gas-gun system located at Korea Institute of Civil Engineering and Building Technology (KICT), shown in Figure 1a, was utilized for the high-speed impact test. The gas-gun is comprised of a high-pressure chamber that can pressurize nitrogen gas to the working pressure 2000 psi and a 40 mm diameter gun-barrel. The gas-gun system is fabricated to a vacuum chamber that has the inner diameter 1000 mm and length 1485 mm, and the jig frame to fix an aluminum target is located in the chamber. The aluminum plate was installed to the steel frame set in the vacuum chamber as shown in Figure 1b, and the projectiles fired through the gas-gun by releasing gas pressure momentarily. The material properties of the 7075-T651 aluminum are summarized in Table 1. The gas-gun is capable of propelling a 200 g projectile at speed up to 400 m/s. The projectile set consists of a warhead made of steel and a sabot made of polycarbonate, as shown in Figure 1c. The steel warhead has a diameter of 36 mm and a thickness of 15 mm and weighs 120 g. The sabot has a groove in the shape of a cylinder on the front face to mount the warhead, and is made of 40 mm in diameter and 80 mm in length so that it can be fired through the barrel. In order to minimize the influence of various variables of geometric shape, such as nose and projectile shapes, the cylindrical projectile, which is the simplest form, is used for the impact tests. Each aluminum target plate was installed to the jig frame in the vacuum chamber, and the movement of the projectile propelled from the gas-gun was recorded through the side window using a high-speed camera (Phantom V711, Vision Research, NJ, USA). The videos were recorded at 20,000 fps with a resolution of 1088 by 400 in grayscale.

### 3.2. Impact Tests on 7075-T651 Aluminum Plates

Impact tests using the gas-gun system were carried out on 7075-T651 aluminum plates of 400-mm width, 400-mm height, and 5- or 10-mm thicknesses, respectively. We performed 5 impact tests for each target thickness with different impact velocities. Initial and residual velocities are measured by observing the travel distance of the projectile per each frame using images captured from the high-speed camera. The initial velocity was measured as 152.2 m/s when the projectile was propelled with the gas pressure of 120 psi, and by increasing the gas pressure to 1500 psi, the initial velocity increased to 372.8 m/s.

**Figure 2:**
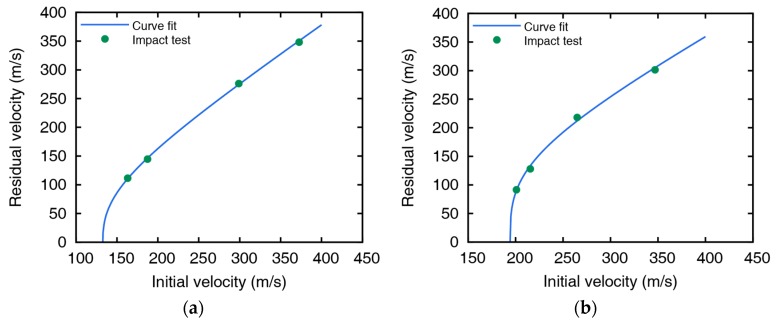
Initial and residual velocities with curve fit for (**a**) 5 and (**b**) 10 mm thick aluminum plates.

**Figure 3:**
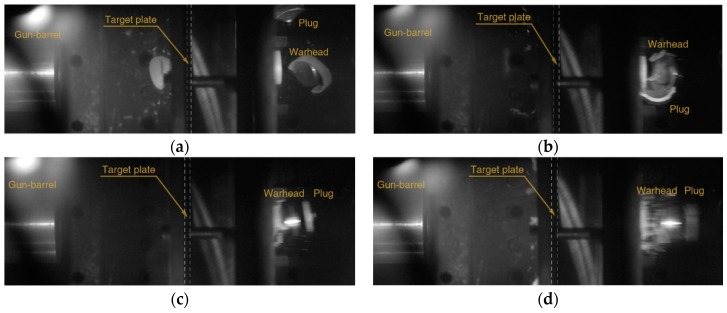
Perforations through 5 mm 7075-T651 plates showing the residual velocities of (**a**) 111.2, (**b**) 144.4, (**c**) 276.2, and (**d**) 348.3 m/s by projectiles at the initial velocities of (**a**) 163.2, (**b**) 187.4, (**c**) 298.8, and (**d**) 372.8 m/s.

**Figure 4:**
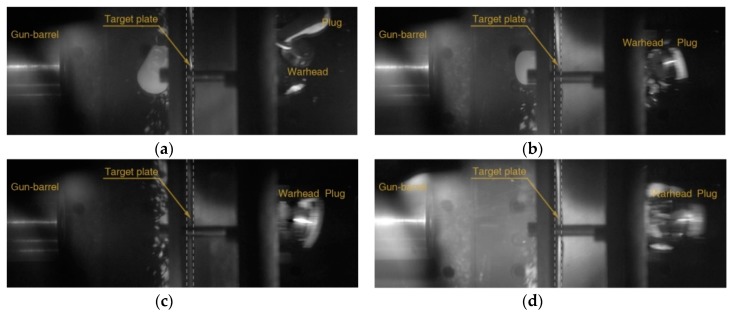
Perforations through 10 mm 7075-T651 plates showing residual velocities of (**a**) 91.5, (**b**) 128.2, (**c**) 218.1, and (**d**) 301.2 m/s by projectiles at initial velocities of (**a**) 200.9, (**b**) 215.5, (**c**) 264.9, and (**d**) 346.8 m/s.

Experimental results are fitted to a model suggested by Lambert [30] to represent the residual velocity of the projectile as a function of impact velocity, as shown in Figure 2. The ballistic limits are obtained as 132.7 and 194.2 m/s for 5 and 10 mm thickness plates, respectively. The images after perforation captured by the high-speed camera are shown in Figure 3 and Figure 4, and the measured initial and residual velocities are summarized in Table 2 and Table 3. Videos of the impact tests are provided as Supplementary materials of Video S1–S8.

The polycarbonate sabot was destroyed at the front surface of the target during the collision, and only the steel warhead breaks through the target and proceeds toward the rear side in the case of perforation. The perforation failure modes were observed at the impact velocity of equal or more than 163.2 m/s for 5 mm thickness and 200.9 m/s for 10 mm thickness, and the penetration mode occurs at the lowest velocity for each thickness (127.0 m/s for 5 mm thickness and 150.6 m/s for 10 mm thickness).

## 4. Numerical Simulations

### 4.1. Numerical Model and Impact Simulations

In order to investigate the impact tests numerically, finite element simulations are carried out. With a nonlinear finite element program, LS-DYNA, projectiles and target plates are discretized with 8-node solid elements. The plastic-kinematic model and the simplified Johnson-Cook model [29,31] are used as material models for the cylindrical projectile and targets, respectively. Because projectiles had little deformation during the collision, the projectile is constructed by a simple plastic material model which has a Young’s modulus of 200 GPa, Poisson’s ratio of 0.3, and yield stress of 710 MPa. The material constants in Equation (1) are listed in Table 4 by referring to the results in [32]. The projectile set consists of a steel warhead and a polycarbonate sabot in the test, whereas only the steel warhead is modeled for the numerical simulation. To make the projectile mass quantity identical to the experiment, the total mass of the projectile set is given to the numerical model of the steel warhead by modifying the mass density.

**Figure 5:**
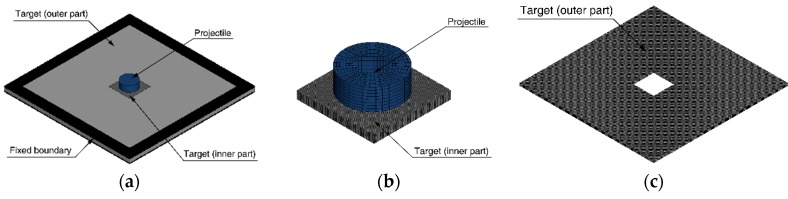
Numerical models of (**a**) projectile and target plate, (**b**) inner part, and (**c**) outer part of the target plate.

We use the 8-node solid elements for all of the numerical models with the constant stress solid element formulation. The projectile is discretized with 2592 solid elements, and the number of elements for the target plate model is summarized in Table 5 and Table 6. The targets are discretized with different sizes of elements to investigate the effect of the element size on impact simulations. To alleviate the computational cost, the inner part of the target (Figure 5b) is modeled with fine elements and is attached to the outer part (Figure 5c) discretized with coarse elements (element size is 1.25 mm). The locations of the nodes in the inner and outer parts are not identical because the element size of the inner part varies. Therefore, the surface to surface contact condition [33] is used to attach the inner part to the outer part regardless of the nodal position. The element size of the inner part is considered as 0.31, 0.50, 0.63, 0.83, and 1.25 mm to include 4 to 16 elements in the thickness direction for the 5 mm-thick plates and 8 to 32 elements for 10 mm-thick plates. A total of 40 numerical models are constructed considering two thicknesses of the plates, four impact velocities for each thickness, and five element sizes. In order to describe the boundary condition of the plate mounted in the frame jig, all nodes in the area where the plate and the jig meet are constrained. The area is 2 cm from the outer edge of the plate and is shown in Figure 5a. The required time step is determined by the material properties and the size of the element and is constantly updated during the simulation as the material deforms [33]. All simulations are set to use the value of 0.9 times the required time step for stable analysis.

**Figure 6:**
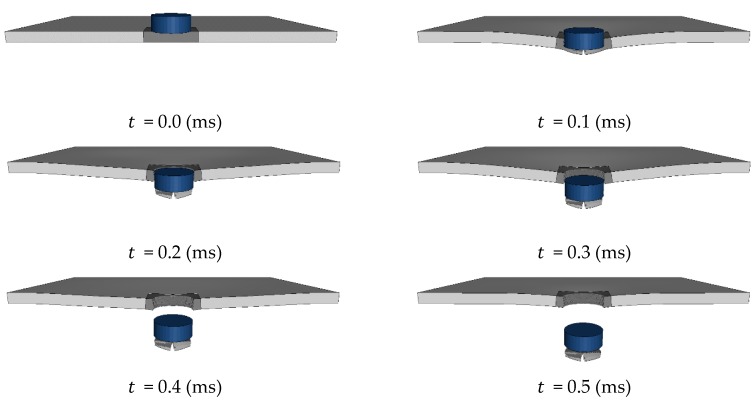
Impact response of a 10 mm thick aluminum plate by a projectile with an initial velocity of 200.9 m/s with the element size of 0.63 mm.

Figure 6 shows the result of numerical analysis where the projectile collides with a 10 mm thick aluminum plate at an initial velocity of 200.9 m/s, and the simulation takes 1080 s with the element size of 0.63 mm. The physical behavior of the perforation process is represented by numerical simulations. In the first step, the overall bend of the plate occurs as the projectile pushes the target in the direction of impact. As the mass in front of the projectile is accelerated by the projectile and the elements near the shear area are damaged, the elements beyond the critical failure strain are removed, and the formed plug is separated from the target. As the projectile penetrates the target, the elements of the plate are eroded in a circular shape.

### 4.2. Failure Strain Value for Residual Velocity

**Figure 7:**
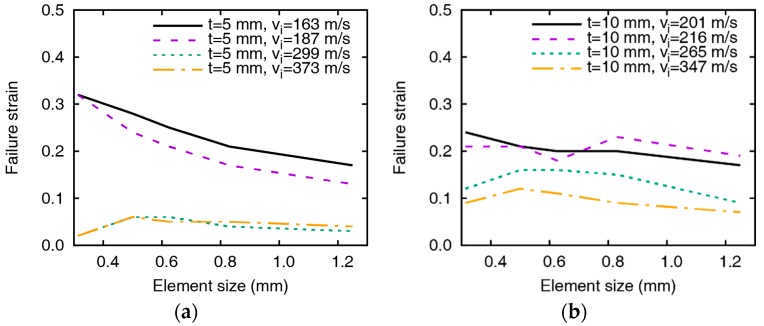
Failure strain by the change of element size. (**a**) $t_{\mathrm{pl}}$ = 5 mm and (**b**) $t_{\mathrm{pl}}$ = 10 mm.

**Figure 8:**
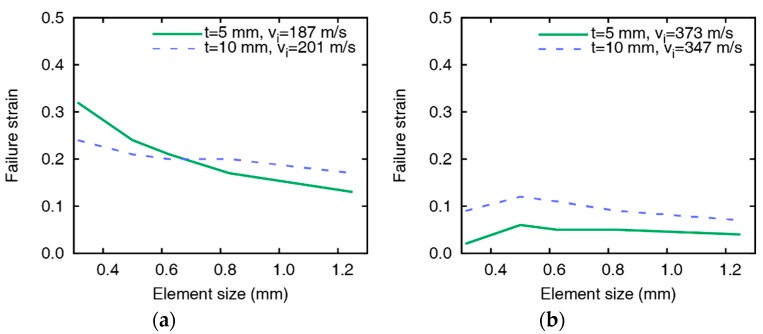
Comparison of failure strains at similar impact velocities for the plates of thicknesses 5 mm and 10 mm. Failure strins by (**a**) impact velocities at 187.4 and 200.9 m/s and (**b**) impact velocities at 346.8 and 372.8 m/s.

The values of the failure strain are obtained through iterative simulations so that the residual velocities are identical with the test results. The changes in the failure strain depending on element size, impact velocity, and plate thicknesses are summarized in Figure 7. From the variation of the failure strain, it is found that the failure strain is inversely proportional to the element size and impact velocity. The value of failure strain for simulating accurate residual velocity gradually decreases as the element size, and the impact velocity increases and is sensitive to small element and low impact velocity. However, the failure strain hardly changes even when the element size varies if the impact velocity is higher than 250 m/s. In the comparison of the failure strains between the 5 mm and 10 mm-thick targets with similar impact velocities shown in Figure 8, the failure strain varies similarly regardless of the thickness of the plate. The failure strain significantly decreases by increasing the element size at relatively low velocities, as shown in Figure 8a, and the failure strain decreases as the impact velocity increases. The change of the failure strain is relatively sensitive if the element is very small, which implies that the failure strain should be carefully handled when using small elements.

### 4.3. The Relationship Between Element Size and Failure Strain with Various Impact Velocity

**Figure 9:**
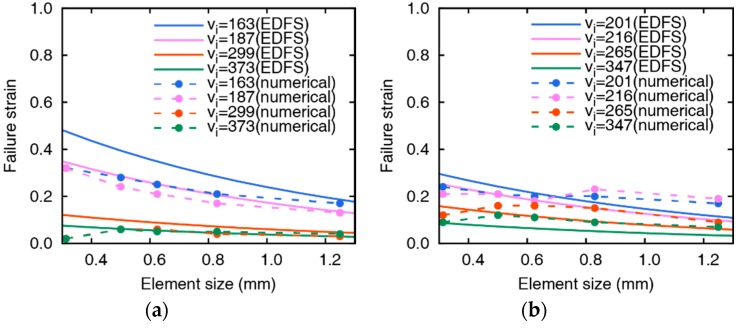
Comparison of numerically obtained failure strain and damage model ($t_{\mathrm{pl}}$ = (**a**) 5 mm and (**b**) 10 mm, *v*_ref_ = 110.0 m/s.).

Figure 9 represents the comparison between failure strain values from iterative simulations and Equation (3). The reference velocity $v_{\mathrm{ref}}$ in Equation (3) is determined as 110.0 m/s that minimizes the error for all eight cases, and the reference element size $h_{\mathrm{ref}}$ is determined as the unit length in mm scale (1 mm). The curves from Equation (3) adequately express the tendency of the failure strain depending on the element size and impact velocity variation. The analytical and numerical values are in good agreement in all eight cases considering both thicknesses and impact velocities. The difference between the analytical value and the numerical value is relatively large because the change of the failure strain caused by the change of the element size is more sensitive in the case of low impact velocity. Although there are slight differences in the low-velocity region, the analytical and numerical values tend to agree well at different thicknesses and various collision velocities.

## 5. Implementation of EDFS to UMAT

### 5.1. Implementation of EDFS in Johnson-Cook Constitutive Model

**Figure 10:**
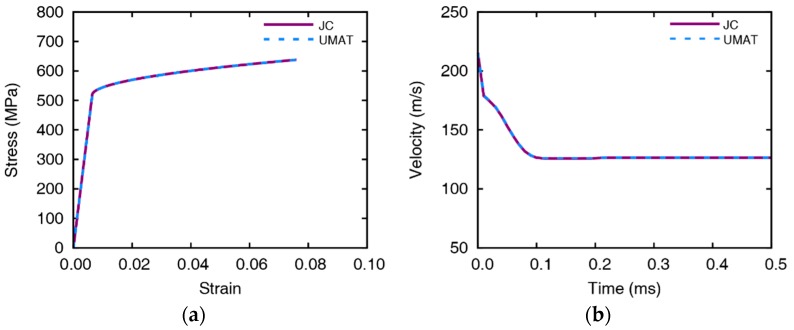
Comparison between the Johnson-Cook model and UMAT: (**a**) stress-strain curves of the tensile simulation and (**b**) residual velocities of the impact simulation with a 10 mm thick aluminum plate and 264.9 m/s of the initial velocity.

We implement the constitutive relationship of the Johnson-Cook material model in the UMAT. The material failure criterion, EDFS, is defined in the UMAT subroutine during the impact simulation by taking the element size and the impact velocity as input variables. In order to verify the UMAT subroutine, tension and impact simulations are performed for comparison with existing Johnson-Cook model. As a result of comparing the stress-strain curves of the tensile simulation, the Johnson-Cook model is simulated to be perfectly matched with UMAT as shown in Figure 10. In the impact simulation with a 5 mm thick aluminum plate and the impact velocity of 215.5 m/s, the time histories of residual velocities almost identical in both cases, and residual velocities are 126.4 and 126.6 m/s for Johnson-Cook model and UMAT, respectively.

### 5.2. Comparative Studies with Johnson-Cook Damage Model

We verify the effectiveness and efficiency of the failure criteria, EDFS, through the comparative study between EDFS and Johnson-Cook damage model. Impact simulations are carried out for two thicknesses of aluminum plate and four impact velocities per each thickness, and the accuracy of the solution and computational efficiency are investigated. The parameters for the Johnson-Cook damage model are determined as $D_{1}\;=\;0.096$, $D_{2}\;=\;0.049$, $D_{3}\;=\;3.465$, $D_{4}\;=\;0.016$, and $D_{5}\;=\;1.099$ by referring to the previous study [32]. The value of $v_{\mathrm{ref}}$ in EDFS is 110 m/s, which is determined from the parametric study in Section 4.3.

**Figure 11:**
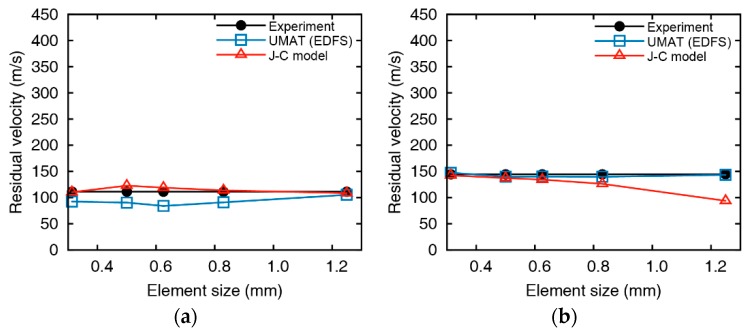

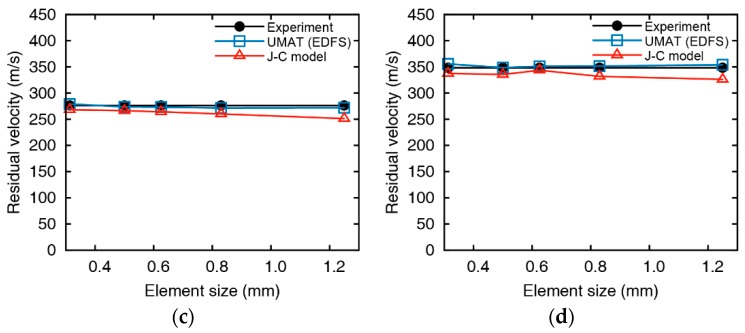
Comparison between impact test and numerical analysis at an initial velocity of (**a**) 163.2, (**b**) 187.4, (**c**) 298.8, and (**d**) 372.8 m/s ($t_{\mathrm{pl}}$ = 5 mm).

**Figure 12:**
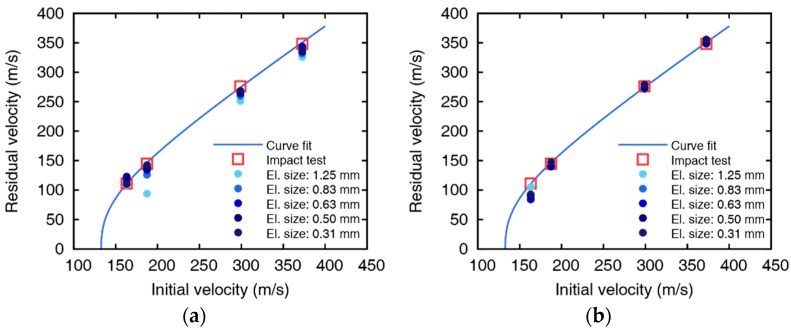
Initial and residual velocities of (**a**) Johnson-Cook damage model and (**b**) EDFS with the curve fit from the impact tests of 5 mm aluminum plates.

The residual velocities of the impact simulations for 5 mm aluminum plates using the EDFS and Johnson-Cook damage model are summarized in Figure 11 and Figure 12. Impact simulations using both the Johnson-Cook model and EDFS predict the residual velocity over the entire range. However, the error of the residual velocity from the Johnson-Cook model increases as the element size increases, and the velocity is predicted to be lower than the impact tests. In the case of low impact velocity ($v_{i}$ = 163.2 m/s), the error of the EDFS model is relatively large compared to the Johnson-Cook model. The reason is that when the impact velocity is not significantly higher than the ballistic limit, the residual velocity varies sensitively with the failure strain. In the other cases except for the low impact velocity, the EDFS model predicts the residual velocity much more accurately than the Johnson-Cook model, and the effect of element size is also much smaller.

**Figure 13:**
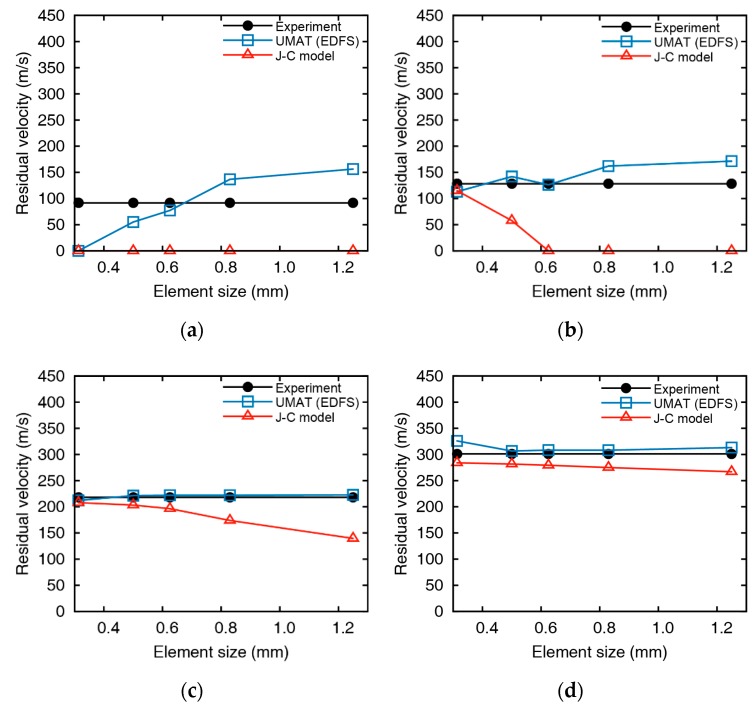
Comparison between impact test and numerical analysis at an initial velocity of (**a**) 200.9, (**b**) 215.5, (**c**) 264.9, and (**d**) 346.8 m/s ($t_{\mathrm{pl}}$ = 10 mm).

**Figure 14:**
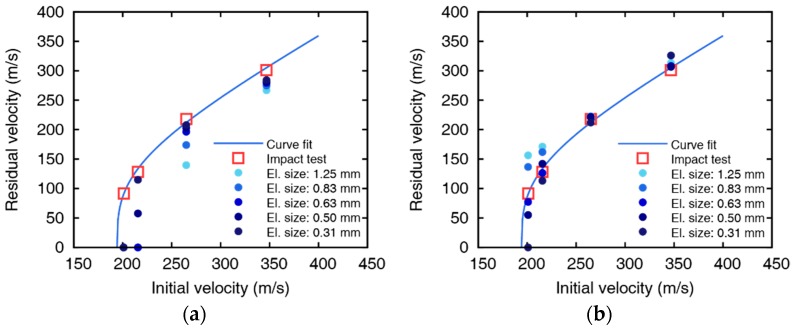
Initial and residual velocities of (**a**) Johnson-Cook damage model and (**b**) EDFS with the curve fit from the impact tests of 10 mm aluminum plates.

Figure 13 and Figure 14 represents the comparison between EDFS and Johnson-Cook model for 10 mm thickness plates. The residual velocity from Johnson-Cook model shows significant differences with the impact test at low impact velocity ($v_{i}$ = 200.9 m/s), and even the failure mode is contradictory with the test. As the impact velocity increases, the accuracy of the Johnson-Cook model improves, but the residual velocities are underestimated in the overall range, and the element size dependency remains. On the other hand, the numerical results of EDFS are hardly affected by the element size in the impact simulations for the 10 mm thick plates. EDFS predicts the residual velocity more precisely than the Johnson-Cook model over the entire range and very accurately predicts the residual velocity, especially in the high impact velocity range. In summary, the EDFS model predicts the relative accuracy of the accurate residual velocity regardless of the element size, while the Johnson-Cook damage model tends to underestimate the residual velocity and shows element size dependent results.

**Figure 15:**
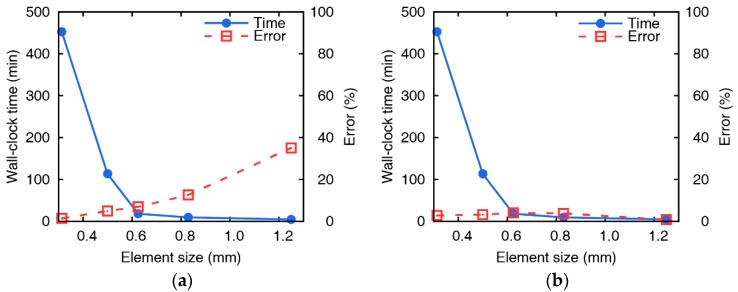
Wall-clock time and residual velocity error versus element sizes using (**a**) the Johnson-Cook damage model and (**b**) the EDFS ($v_{i}\;=\;163.2\;m/s$ and $t_{\mathrm{pl}}\;=\;5\;\mathrm{mm}$).

One of the impact cases is selected to evaluate the effectiveness of the Johnson-Cook model and EDFS by comparing the computation time and accuracy with element size changes as shown in Figure 15. The Johnson-Cook model accurately predicts the residual velocity of a collision experiment when the element size is small enough, but the error increases significantly compared to the EDFS when the element size exceeds 0.6 mm. The Johnson-Cook model requires the element size of smaller than 0.6 mm in order to have an error of smaller than 5%, but the EDFS model ensures sufficient accuracy regardless of the element size. Thus, using the EDFS model, numerical analyses are performed much more efficiently while ensuring accuracy using large size of elements. To predict the residual velocity in error by less than 5%, the Johnson-Cook model takes 6798 s in wall-clock time solved in parallel with 4 threads by using the element size of 0.5 mm. On the other hand, the EDFS model predicts the residual velocity in error by less than 5% using only the calculation time of 270 s solved in parallel with 4 threads using the 1.25 mm of the element size. A workstation which has Dual Intel ® Xeon(R) CPU E5-2687W v2 @ 3.40 GHz of 32 threads and 64 GB memory was used to perform parallel processing. Using the EDFS, it is possible to predict the residual velocity at the same accuracy with only 4% of the computational cost compared to the Johnson-Cook damage model. Thus, numerical results using the Johnson-Cook model requires a sufficiently small element size to ensure accuracy, but the computational cost increases dramatically. If the EDFS model is used, it is possible to predict the accurate residual velocity even with a small computational cost.

## 6. Summary and Conclusions

In the finite element analysis, the accuracy of the numerical solution depends on the element size, and significant computational cost is essential to attain sufficient accuracy. For efficient and accurate impact simulation, we propose an enhanced damage criterion. This criterion can alleviate computational costs in impact simulations while providing more accurate results than existing damage criteria, and we verify the criterion by comparing the numerical results with the impact tests. Also, the damage criterion is combined with the Johnson-Cook constitutive relationship and implemented in UMAT of LS-DYNA for the usability of the damage criterion. Using a gas-gun system, impact tests were carried out to investigate the impact response of the 7075-T651 aluminum plate, and the test results are used as the reference data to verify the numerical model and damage criterion. Numerical models are constructed using various element sizes in order to evaluate the effect of the element size on the impact simulation. The correlations among the failure strain, the impact velocity, and the element size are inversely obtained from numerical simulations by comparing the residual velocities with the test results. It is found that the failure strain varies inversely with the element size and impact velocity. In particular, the sensitivity of the failure strain to the impact velocity significantly increases as the element size decreases and, therefore, the failure strain should be carefully determined when a small element size is used. By applying the characteristic of the failure strain, which depends on the impact velocity and element size, we have introduced an element-size dependent failure strain (EDFS) and the results show good agreement with experimental results regardless of the element size. To import EDFS in the subroutine of the material model, the Johnson-Cook constitutive relationship is implemented in UMAT and the numerical results using UMAT are in good agreement with the existing Johnson-Cook material model. Then, EDFS is imported in UMAT to implement a material model that combines EDFS and Johnson-Cook configuration relationships. When using the Johnson-Cook damage model, the accurate solution is obtained if we use a sufficiently small element size, but the computational cost exponentially increases accordingly. The application of the EDFS allows the calculation time to be reduced significantly because the numerical results are in good agreement with the results of experiments even if the large element size is used. Using the damage criterion presented in this study, efficient simulations can be carried out, ending up with a high accuracy as obtained without very fine discretization.

## Figures and Tables

**Table 1:** Material properties of 7075-T651 aluminum.

Density	Young’s Modulus	Poisson’s Ratio	Yield Strength	Ultimate Strength
2700 kg/m^3^	70 GPa	0.3	520 MPa	572 MPa

**Table 2:** Initial and residual velocities of projectiles ($t_{\mathrm{pl}}$ = 5 mm).

Case	1	2	3	4	5
Initial Velocity (m/s)	127.0	163.2	187.4	298.8	372.8
Residual Velocity (m/s)	-	111.2	144.4	276.2	348.3

**Table 3:** Initial and residual velocities of projectiles ($t_{\mathrm{pl}}$ = 10 mm).

Case	1	2	3	4	5
Initial Velocity (m/s)	150.6	200.9	215.5	264.9	346.8
Residual Velocity (m/s)	-	91.5	128.2	218.1	301.2

**Table 4:** Parameters for simplified Johnson-Cook model for 7075-T651 aluminum plate.

Parameter	*a*	*b*	*n*	*c*
Value	5.20 × 10^8^	4.77 × 10^8^	0.52	0.001

**Table 5:** The number of elements for 5 mm plate model.

Component	Outer Part	Inner Part				
**Element Size (mm)**	1.25	0.31	0.50	0.63	0.83	1.25
**Number of Elements**	22,400	409,600	100,000	51,200	21,600	6400

**Table 6:** The number of elements for 10 mm plate model.

Component	Outer Part	Inner Part				
**Element Size (mm)**	1.25	0.31	0.50	0.63	0.83	1.25
**Number of Elements**	44,800	819,200	200,000	102,400	43,200	12,800

## Supplementary Materials

The following are available online at https://www.mdpi.com/1996-1944/12/17/2736/s1, Video S1: Perforation through 5 mm 7075-T651 plate showing the residual velocity of 111.2 m/s by projectiles at the initial velocity of 163.2 m/s, Video S2: Perforation through 5 mm 7075-T651 plate showing the residual velocity of 144.4 m/s by projectiles at the initial velocity of 187.4 m/s, Video S3: Perforation through 5 mm 7075-T651 plate showing the residual velocity of 276.2 m/s by projectiles at the initial velocity of 298.8 m/s, Video S4: Perforation through 5 mm 7075-T651 plate showing the residual velocity of 348.3 m/s by projectiles at the initial velocity of 372.8 m/s, Video S5: Perforation through 10 mm 7075-T651 plate showing the residual velocity of 91.5 m/s by projectiles at the initial velocity of 200.9 m/s, Video S6: Perforation through 10 mm 7075-T651 plate showing the residual velocity of 128.2 m/s by projectiles at the initial velocity of 215.5 m/s, Video S7: Perforation through 10 mm 7075-T651 plate showing the residual velocity of 218.1 m/s by projectiles at the initial velocity of 264.9 m/s, Video S8: Perforation through 10 mm 7075-T651 plate showing the residual velocity of 301.2 m/s by projectiles at the initial velocity of 346.8 m/s.
